# Progress of medicinal plants and their active metabolites in ischemia-reperfusion injury of stroke: a novel therapeutic strategy based on regulation of crosstalk between mitophagy and ferroptosis

**DOI:** 10.3389/fphar.2024.1374445

**Published:** 2024-04-08

**Authors:** Guozhen Zhang, Qiang Wang, Bing Jiang, Lihe Yao, Wenjuan Wu, Xiaoyan Zhang, Dongjun Wan, Youquan Gu

**Affiliations:** ^1^ College of the First Clinical Medicine, Lanzhou University, Lanzhou, Gansu, China; ^2^ Department of Neurology, People’s Liberation Army Joint Logistics Support Force 940th Hospital, Lanzhou, Gansu, China; ^3^ Department of Neurology, First Hospital of Lanzhou University, Lanzhou, Gansu, China; ^4^ Department of Integrated Chinese and Western Medicine, Gansu University of Traditional Chinese Medicine, Lanzhou, Gansu, China

**Keywords:** mitophagy, ferroptosis, ischemia-reperfusion injury in stroke, medicinal plants, active metabolites

## Abstract

The death of cells can occur through various pathways, including apoptosis, necroptosis, mitophagy, pyroptosis, endoplasmic reticulum stress, oxidative stress, ferroptosis, cuproptosis, and disulfide-driven necrosis. Increasing evidence suggests that mitophagy and ferroptosis play crucial regulatory roles in the development of stroke. In recent years, the incidence of stroke has been gradually increasing, posing a significant threat to human health. Hemorrhagic stroke accounts for only 15% of all strokes, while ischemic stroke is the predominant type, representing 85% of all stroke cases. Ischemic stroke refers to a clinical syndrome characterized by local ischemic-hypoxic necrosis of brain tissue due to various cerebrovascular disorders, leading to rapid onset of corresponding neurological deficits. Currently, specific therapeutic approaches targeting the pathophysiological mechanisms of ischemic brain tissue injury mainly include intravenous thrombolysis and endovascular intervention. Despite some clinical efficacy, these approaches inevitably lead to ischemia-reperfusion injury. Therefore, exploration of treatment options for ischemic stroke remains a challenging task. In light of this background, advancements in targeted therapy for cerebrovascular diseases through mitophagy and ferroptosis offer a new direction for the treatment of such diseases. In this review, we summarize the progress of mitophagy and ferroptosis in regulating ischemia-reperfusion injury in stroke and emphasize their potential molecular mechanisms in the pathogenesis. Importantly, we systematically elucidate the role of medicinal plants and their active metabolites in targeting mitophagy and ferroptosis in ischemia-reperfusion injury in stroke, providing new insights and perspectives for the clinical development of therapeutic drugs for these diseases.

## 1 Introduction

As a common and severe neurological systemic disease, stroke can be categorized into ischemic stroke and hemorrhagic stroke. Globally, the incidence of both types of stroke is increasing annually, leading to a significant rise in mortality and disability, especially in low- and middle-income countries. Hemorrhagic stroke accounts for only 15% of all strokes, while ischemic stroke is the predominant type, representing 85% of all strokes (Feske, 2021; Montaño et al., 2021). Ischemic stroke refers to a clinical syndrome characterized by local ischemic and hypoxic necrosis of brain tissue due to various cerebrovascular lesions, leading to rapid onset of corresponding neurological deficits. Currently, specific therapeutic approaches for the pathophysiological mechanisms of ischemic brain tissue injury mainly include intravenous thrombolysis and endovascular intervention therapy (Rabinstein, 2020). Although these methods have shown some clinical efficacy, they inevitably lead to ischemia-reperfusion injury. Therefore, it is an urgent global need to explore the comprehensive pathogenic mechanisms and effective diagnostic and therapeutic methods for ischemic stroke. Increasing evidence suggests that ischemia-reperfusion injury is an important link in the pathogenesis of ischemic stroke, and in this process, the regulation of cellular physiological processes such as mitophagy and ferroptosis becomes particularly crucial (Liu et al., 2013; Zhang et al., 2022a; She et al., 2023). Ischemia-reperfusion injury in cerebral strokeresults from cellular damage due to insufficient cerebral blood flow, exacerbated by oxidative stress and inflammatory reactions during reperfusion. Mitophagy and ferroptosis, as important intracellular regulatory mechanisms, have received increasing attention from scholars in recent years. Activation of mitophagy during the process of ischemic stroke reperfusion injury helps clear dysfunctional mitochondria, alleviate oxidative stress, and exert a protective effect. Studies have shown that by regulating the signaling pathways related to mitophagy, it is possible to alleviate neuronal cell damage caused by ischemic stroke reperfusion and thus slow down the progression of stroke (Zhou et al., 2015; Hwang et al., 2023). Additionally, ferroptosis, as a novel form of cell death caused by the accumulation of intracellular iron ions, is closely related to the release and accumulation of local iron ions in ischemic stroke reperfusion injury. Inhibiting ferroptosis can alleviate cell membrane damage and neuroinflammatory reactions, offering new insights into the treatment strategies for stroke (Liu et al., 2023c; 2023b).

Cell death serves as a vital component of the entire cell life cycle and is essential for individual growth, development, and survival. Cell death occurs regularly in normal tissues and is necessary to maintain metabolic balance. Previous studies have shown that cell death pathways can be largely classified into two classic forms: apoptosis and necrosis (Kopeina and Zhivotovsky, 2022). Apoptosis is a highly regulated form of programmed cell death that can occur during multicellular organism development, throughout the life cycle, and in response to cellular stress. Apoptosis is primarily mediated by the “protease-rich” Caspase family. In addition, other proteins (including pro-apoptotic and anti-apoptotic proteins) also play essential roles (Fleisher, 1997). Dysregulation of cell apoptosis has been observed in various disease states (including autoimmune diseases, neurodegenerative diseases, and cancer) (Giovannetti et al., 2008; Wong, 2011; Erekat, 2022). Necrosis is a type of non-programmed cell death that mainly occurs in acute injury, infection, and when apoptosis is inhibited, characterized by cell swelling and dissolution. Pathologically, necrotic cells can release cell contents into the surrounding environment and recruit phagocytes to clear dead cells through triggering inflammation. However, uncontrolled necrosis can also cause severe tissue damage, such as gangrene (Festjens et al., 2006). In recent years, with the continuous development of molecular biology techniques, numerous novel forms of cell death have emerged, such as mitophagy (Lu et al., 2023a), ferroptosis (Dixon and Pratt, 2023), cuproptosis (Kahlson and Dixon, 2022), disulfide-driven necrosis (Liu et al., 2023d), and so on.

The concept of mitophagy was proposed by Lemasters in 2005. Under stressors such as reactive oxygen species (ROS) exposure, mitochondrial DNA mutations accumulate, leading to decreased mitochondrial membrane potential and depolarization damage. This ultimately results in cell death. In this severe survival situation, mitochondria have to “kill” to maintain mitochondrial and cellular homeostasis and prevent damaged mitochondria from causing further harm to the cell. To do so, cells selectively encapsulate and degrade damaged or dysfunctional mitochondria within the cell. Mitophagy can be divided into four key steps: (1) Depolarization of damaged mitochondria, resulting in loss of membrane potential. (2) Mitochondria are encapsulated by autophagosomes to form mitophagosomes. (3) Mitophagosomes fuse with lysosomes. (4) The contents of mitochondria are degraded by lysosomal enzymes, and acidic hydrolases from lysosomes or vacuoles flow into the autophagosomes to degrade damaged mitochondria. Increasing research suggests that selective clearance of dysfunctional mitochondria through mitophagy plays a crucial role in maintaining mitochondrial quantity, quality homeostasis, and cell survival (Lu et al., 2023b).

The concept of ferroptosis was first proposed by Dixon *et al.* in 2012, mainly used to describe the cell death mechanism induced by Earstin, characterized by the inhibition of intracellular cysteine transport, leading to glutathione consumption and inactivation of glutathione peroxidase 4 (GPX4) (Tang et al., 2021). Unlike apoptosis, autophagy, and necrosis, ferroptosis is a form of programmed cell death regulated by iron ions, characterized by increased intracellular iron ions, accumulation of lipid peroxides and related metabolic products, and peroxidation of polyunsaturated fatty acids in the plasma membrane. Morphologically, ferroptosis involves significant shrinkage of mitochondria, increased density of double-layer membrane structure, and loose or disappeared cristae formation. Biochemically, GPX4 activity decreases, excessive glutathione consumption, lipids in the cell are oxidized by divalent iron ions in a manner similar to the Fenton reaction, resulting in the production of large amounts of ROS. Genetically, ferroptosis is associated with abnormal expression of multiple genes, particularly those related to iron regulation, including RPL8, IREB2, ATP5G3, CS, TTC35, ACSF2, and so on (Hadian and Stockwell, 2020). It is noteworthy that with the continuous development of molecular biology techniques, regulation of this type of cell death has been extensively studied. In numerous cell experiments, ferroptosis inducers have been confirmed to mainly belong to the RAS selective killing factor family, represented by Earstin and RSL3. Inhibitors have been developed relatively more extensively, including classic ferrostatin-1, iron chelator deferoxamine, antioxidant Trolox, mitogen extracellular signal-regulated kinase (ERK) inhibitor U0126, and so on (Zeng et al., 2023). Thus, these ferroptosis-related regulators are expected to become a new direction for disease prevention and treatment.

In fact, there is a close relationship between mitophagy and ferroptosis, as they both participate in the regulation of cell function and intracellular homeostasis. On one hand, excessive free iron can lead to mitochondrial dysfunction, thereby triggering mitophagy. The goal of mitophagy is to package damaged mitochondria into autophagosomes and degrade them through lysosomes, thus preventing harmful substances from leaking out of the cell. This process can regulate intracellular iron ion levels to some extent, thereby influencing the occurrence and progression of ferroptosis (Lin et al., 2023). On the other hand, ferroptosis may also have a certain impact on mitophagy. Some studies have shown that during the process of ferroptosis, oxidative stress levels in the cell increase, which may affect mitochondrial stability and initiate mitophagy (Li et al., 2023a). Furthermore, some molecular signaling pathways related to iron metabolism may participate in the regulation of mitophagy (Bi et al., 2024). Overall, the relationship between ferroptosis and mitophagy involves complex signaling pathways and molecular mechanisms. Current research is continuously deepening, and understanding these interactions may help reveal the molecular mechanisms of cell death and cell self-regulation, which may have important implications for the treatment of related diseases. Increasing evidence suggests that mitophagy and ferroptosis play essential roles in the occurrence and development of ischemia-reperfusion injury in stroke (Li et al., 2021b; Shen et al., 2021).

In summary, mitophagy and ferroptosis play important roles in the pathophysiological processes of stroke, providing new avenues for the treatment of stroke and its complications. Therefore, this article systematically reviews the mechanisms of mitophagy and ferroptosis. Furthermore, we have studied their roles in ischemic stroke reperfusion injury. Importantly, this article also systematically elucidates the roles of medicinal plants and their active metabolites in targeting mitophagy and ferroptosis in ischemic stroke reperfusion injury, providing new directions and insights for the clinical development of therapeutic drugs for such diseases.

## 2 The pathogenesis of mitophagy and ferroptosis

### 2.1 The pathogenesis of mitophagy

**FIGURE 1:**
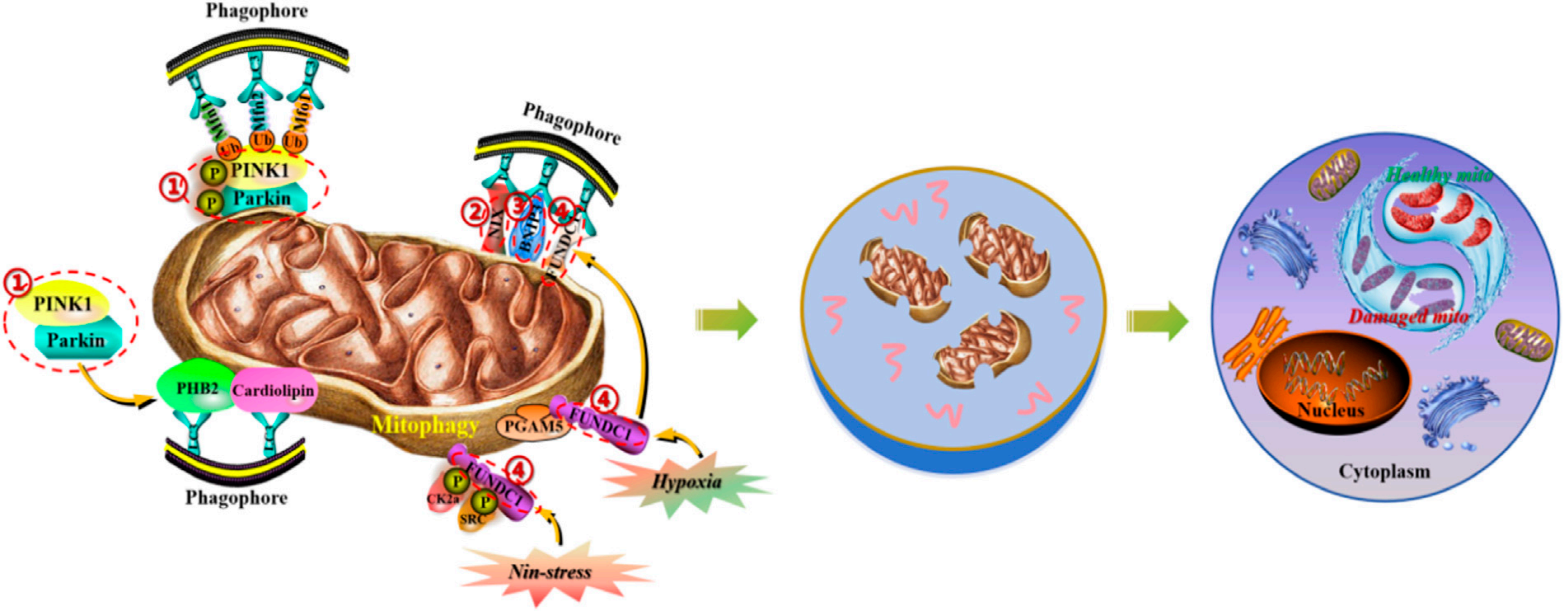
Molecular mechanisms of mitophagy in the maintenance of mitochondrial homeostasis. (1) PINK1/Parkin signaling pathway induces mitochondrial autophagy by regulating the activity of p62/SQSTM1 and LC3 proteins. (2) NIX protein, located on the outer membrane of mitochondria, induces mitochondrial autophagy by directly binding to LC3. (3) BNIP3 protein, as the HIF- α target gene, can synergistically induce mitochondrial autophagy with NIX protein. (4) FUNDC1 protein, located on the outer membrane of mitochondria, induces mitochondrial autophagy by dephosphorylation and binding to LC3.

Mitochondria are dynamic organelles with diverse functions, playing crucial roles in cellular metabolism and survival, including involvement in necrotic cell death and programmed apoptosis. Mitophagy is a cellular protective mechanism that removes excess or dysfunctional mitochondria, maintaining a fine-tuned balance of mitochondria to ensure intracellular homeostasis. There is increasing evidence that mitophagy, as an acute tissue stress response, plays a crucial role in maintaining the health of the mitochondrial network. Due to the critical importance of timely removal of abnormal mitochondria for cell survival, cells have evolved multiple pathways for mitophagy to ensure its timely activation in various environments. A better understanding of the mechanisms of mitophagy in various diseases is crucial for the treatment and design of therapeutic targets for diseases. Here, we summarize the molecular mechanisms mediated by mitophagy, thereby maintaining the dynamic balance of mitochondria at the systemic and organ levels, laying a theoretical foundation for the development of precision medical new treatment strategies based on the research progress of mitophagy signal transduction (Figure 1).

#### 2.1.1 PINK1/Parkin signaling pathway

PINK1 is a protein kinase located in the inner mitochondrial membrane (IMM) that promotes Parkin phosphorylation and facilitates its transfer to mitochondria. When mitochondrial damage occurs and leads to depolarization, PINK1 accumulates significantly, phosphorylating Mfn2 to enhance its binding ability with Parkin, upregulating its autophosphorylation level, or phosphorylating and ubiquitinating Parkin. This promotes Parkin’s transfer from the cytoplasm to mitochondria. Under this condition, Parkin can form a substrate polyubiquitin chain through the E3 ligase complex by interacting with the activated Ub and voltage-dependent anion channel 1 (VDAC1). Subsequently, Parkin can be recognized and bound by the ubiquitin-binding protein p62/SQSTM1 and microtubule-associated protein 1 light chain 3 (LC3), aggregating ubiquitinated proteins in new autophagosomes and fusing with lysosomes to form autophagic lysosomes. These lysosomes degrade and clear damaged mitochondria through acidic hydrolases within the body. As Wang HY *et al.* (Wang et al., 2019) found, electroacupuncture therapy can improve nitro/oxidative stress-induced mitochondrial dysfunction and reduce the accumulation of damaged mitochondria by promoting mitophagy through the Pink1/Parkin-mediated pathway, thereby protecting cells from neuronal damage in brain ischemia-reperfusion. It is noteworthy that the non-dependent mitophagy pathway on Parkin is still under investigation. For example, Szarhel R *et al.* (Szargel et al., 2016) demonstrated that PINK1 can accumulate in the outer mitochondrial membrane (OMM) and recruit SIAH-1 through the mediation of Synphilin-1, leading to ubiquitination of damaged mitochondria and accelerating the autophagic process.

#### 2.1.2 NIX protein

NIX is a protein mainly located in the OMM and belongs to the anti-apoptotic B-cell lymphoma-2 homology domain 3. It can directly bind to LC3 to induce mitophagy. Research has shown that NIX is crucial for mitochondrial clearance during erythrocyte maturation, as mitochondrial depolarization, massive generation of ROS, and hypoxia can induce NIX expression, thereby activating the mitophagic pathway (Wu et al., 2021b; Li et al., 2021c).

#### 2.1.3 BNIP3 protein

BNIP3 is a target gene of hypoxia-inducible factor 1*α* (HIF-1*α*). When ischemic stroke occurs, hypoxic conditions activate HIF-1α, leading to upregulated expression of NIX and BNIP3 in cells, and thus activating the mitophagic pathway. As Fu ZJ *et al.* (Fu et al., 2020) found, knocking out HIF-1α in renal tubules can significantly inhibit ischemia-reperfusion-induced mitophagy, exacerbating renal tubular apoptosis and injury. In contrast, adenoviral-mediated overexpression of BNIP3 significantly reverses the reduction of mitophagy and prevents renal injury enhancement in mice with ischemia-reperfusion injury, indicating a protective role of HIF-1α-BNIP3-mediated mitophagy in renal tubular cells by inhibiting acute kidney injury cell apoptosis and ROS production. Other studies have reported that NIX/BNIP3 is involved in ischemia-reperfusion-induced mitophagy, but excessive upregulation of BNIP3 can lead to cell death, and NIX may only participate in the regulation of the basic level of mitophagy under physiological conditions (Zhang and Ney, 2009).

#### 2.1.4 FUNDC1 protein

FUNDC1 is a trimeric transmembrane protein located in the outer mitochondrial membrane. It exists stably in the outer membrane under normal conditions and does not mediate mitophagy. When mitochondrial damage or dysfunction occurs, the expression levels of FUNDC1 and LC3 increase, and FUNDC1 can induce mitophagy by dephosphorylation. As Zhou H *et al.* (Zhou et al., 2018) discovered, inhibiting mitophagy through the mTORC1-ULK1-FUNDC1 pathway can protect against myocardial ischemia-reperfusion injury, suggesting the potential existence of a similar mechanism in brain ischemia-reperfusion injury.

### 2.2 The pathogenesis of ferroptosis

**FIGURE 2:**
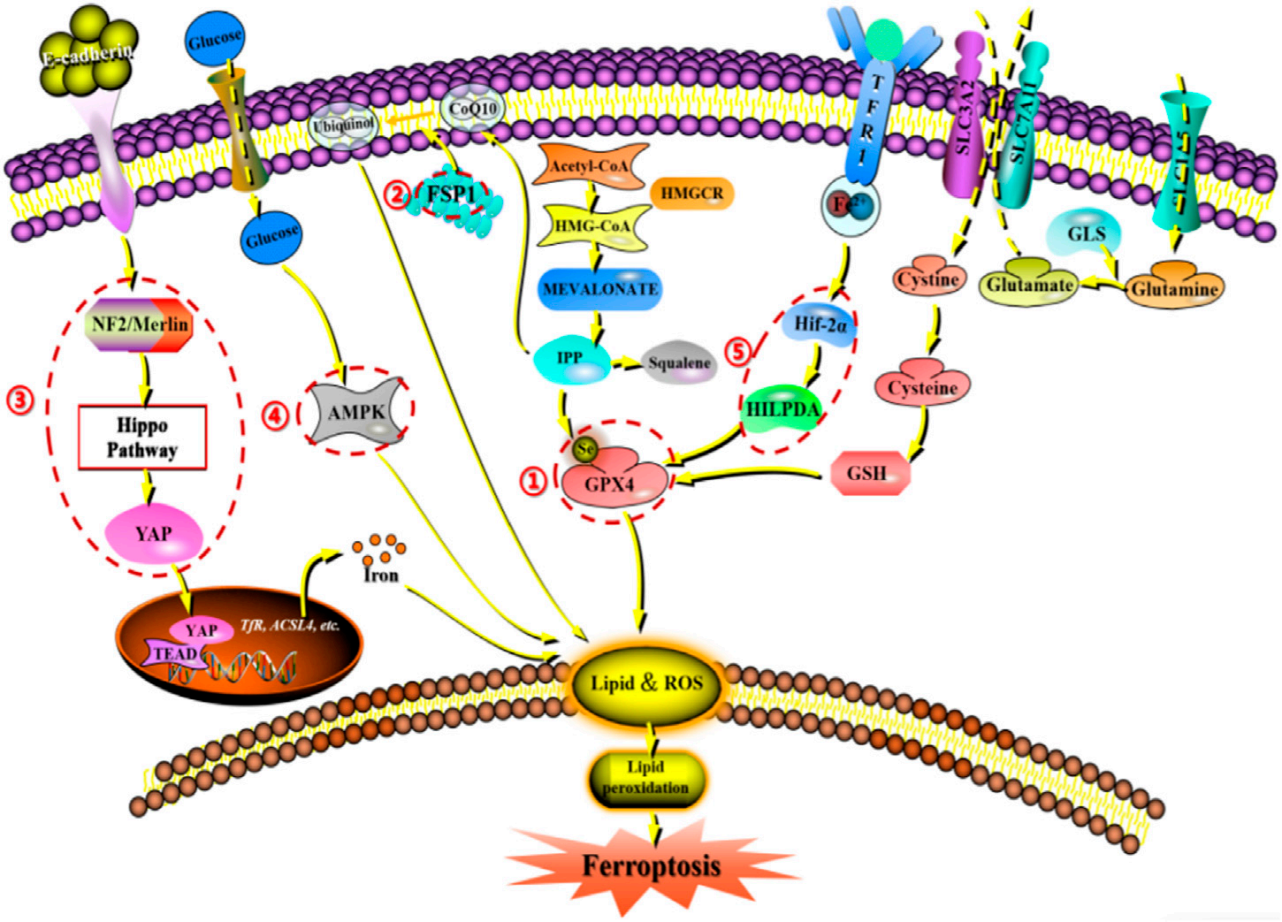
Network crosstalk of molecular mechanisms during ferroptosis development. (1) GPX protein regulates ferroptosis by clearing lipid peroxide products on the membrane. (2) FSPA gene regulates ferroptosis by acting on reducing and oxidizing substrates. (3) E-cadherin/NF2/Hippo/YAP signaling pathway regulates ferroptosis by mediating the activity of ferroptosis regulatory factors. (4) AMPK signaling pathway regulates ferroptosis by initiating an energy stress protective program. (5) HIF-2 α/The HILPDA signaling pathway regulates ferroptosis by affecting phospholipid peroxidation.

Since the description of ferroptosis as an iron-dependent non-apoptotic cell death form in 2012, there has been increasing interest in the process and function of ferroptosis. Ferroptosis can occur through two major pathways: exogenous or transporter-dependent pathways and endogenous or enzyme-regulated pathways. Ferroptosis is caused by an imbalance in the production of oxidants and antioxidants, driven by the abnormal expression and activity of various redox enzymes involved in the generation or detoxification of free radicals and lipid oxidation products. Therefore, ferroptosis is precisely regulated at multiple levels, including the epigenetic, transcriptional, post-transcriptional, and post-translational layers. In this review, we summarize the current knowledge of the comprehensive molecular mechanisms of ferroptosis and describe how dysregulated ferroptosis can participate in the development of diseases (Figure 2).

#### 2.2.1 GPX4 protein

GPX4, also known as phospholipid hydrogen peroxide glutathione peroxidase (PHGPx), is the fourth member of the selenium-containing GPX family. The GPX4 protein has a molecular weight of approximately 19 kDa and is composed of about 170 amino acids. Several GPX family members, including GPX1-GPX8, have been found in mammals. However, only GPX4 exhibits the ability to clear membrane lipid peroxyl radicals, which is closely related to its unique amino acid sequence and spatial structure. Studies have shown that increasing the intracellular selenium content can synergistically activate transcription factors TFAP2c and Sp1 to promote GPX4 expression in neurons. Persistent oxidative stress can cause GSH depletion, which leads to the impairment of the GSH-dependent reduction of the selenocysteine active site in GPX4 and the formation of dehydroalanine and irreversible inactivation of GPX4 through the *β*-cleavage process. In addition, selenamide formed between selenite and nearby amino acids can protect the enzyme from irreversible inactivation. Multiple ferroptosis inducers ultimately lead to GPX4 depletion through covalent modification of the selenocysteine active site, interference with methionine metabolism, and iron-dependent oxidative stress mechanisms (Liu et al., 2023e). Notably, according to a 2014 targeted metabolomics study, overexpression or knockdown of GPX4 can regulate the lethality of 12 ferroptosis inducers to cells (Yang et al., 2014). Thus, GPX4 has been confirmed as a key regulatory factor for ferroptosis. Mechanistically, GPX4 utilizes its catalytic activity to weaken the toxicity of lipid peroxides and maintain the stability of the lipid bilayer. RSL3, as an inhibitor of GPX4, can covalently bind to GPX4 and inactivate it, leading to the accumulation of intracellular peroxides and ultimately triggering ferroptosis (Li et al., 2021a). Furthermore, as a cofactor for GPX4 to convert peroxides to alcohols, the lack of glutathione can cause cysteine deficiency, which will directly inactivate GPX4 and trigger ferroptosis (Ursini and Maiorino, 2020).

#### 2.2.2 FSP1 gene

FSP1 is a target gene of transcription factors NRF2, CRBP, and PPARα. Interestingly, it has been reported that in T-lymphoblastoid lymphoma cells, long non-coding RNA MEG3 (maternally expressed gene 3) upregulates FSP1 expression, while miR-214 downregulates FSP1 expression, and both types of RNA are also involved in tumor development (Brewer, 2023). Increasing evidence indicates that FSP1 expression levels are positively correlated with ferroptosis resistance in hundreds of tumor cell lines and mainly manifest in FSP1-mediated ferroptosis resistance in lung cancer cell culture and mouse xenograft tumors (Hendricks et al., 2023). Therefore, FSP1 expression is crucial for predicting the efficacy of ferroptosis-inducing drugs in cancer, and FSP1 inhibitors have been identified as a potential strategy to overcome ferroptosis resistance in many cancers. In addition to transcriptional regulation, little is known about how the redox enzyme activity of FSP1 is regulated and how its subcellular localization controls its function in different physiological and pathological processes. However, FSP1 can act on both reducing and oxidizing substrates (such as NADH, NADPH, CoQ10, and *α*-tocopherol), indicating that its regulatory process is complex (Lv et al., 2023).

#### 2.2.3 E-cadherin/NF2/Hippo/YAP signaling pathway

The role of the E-cadherin-NF2-Hippo-YAP pathway in regulating ferroptosis is of great significance. The regulation of ferroptosis by cell density in epithelial cells is mediated by E-cadherin-mediated intercellular contact. This intercellular interaction can activate the intracellular Hippo signaling pathway through the tumor suppressor protein NF2 (also known as Merlin), thereby inhibiting the nuclear translocation and transcription activity of tumor protein YAP. As YAP targets multiple ferroptosis regulatory factors, including ACSL4 and transferrin receptor TfR1, the occurrence of ferroptosis ultimately depends on the activity of the Hippo pathway. Inhibition of the Hippo pathway and activation of YAP can accelerate the process of ferroptosis (Wu et al., 2019a; Wang et al., 2022).

#### 2.2.4 AMPK signaling pathway

Energy and metabolic stress cause energy loss, leading to the loss of control of the systemic cascades required to maintain homeostasis, such as energy-dependent transmembrane ion concentration imbalance, ultimately resulting in cell death. In addition, metabolic stress induced by glucose starvation promotes ROS accumulation in cells, indicating that glucose starvation may promote ferroptosis. Interestingly, glucose starvation inhibits cell ferroptosis, a protective effect that often depends on the activity of the energy-sensing kinase AMPK. Therefore, when glucose is deficient, the AMPK pathway can be activated, initiating an energy stress protection program to counteract ferroptosis. This process involves the impairment of PUFA biosynthesis, which is essential for lipid peroxide-induced ferroptosis. Studies have shown that the activation of the energy stress protection program can protect the kidney from ischemia-reperfusion injury. This AMPK-dependent protective mechanism may be the first line of defense against organ damage caused by energy exhaustion, which often accompanies organ injury (Lee et al., 2020).

#### 2.2.5 HIF-2α/HILPDA signaling pathway

It is well-known that ferroptosis is caused by phospholipid peroxidation. The question then arises as to whether the process of ferroptosis is dependent on oxygen concentration. Early experiments showed that Erastin-induced ferroptosis was not affected in a 1% oxygen environment, indicating that hypoxia does not inhibit ferroptosis. However, recent studies have shown that hypoxia can actually increase the sensitivity of cells to ferroptosis, such as in clear cell carcinoma cells that are highly sensitive to GPX4 inhibitor-induced ferroptosis. This sensitivity is mediated by the HIF-2*α* subtype through the induction of hypoxia-inducible lipid droplet-associated proteins (HILPDA) andPUFA lipid remodeling (Gao et al., 2023). Therefore, it is speculated that the HIF-2*α*/HILPDA signaling pathway-driven sensitivity to ferroptosis in cells may represent a novel method for eliminating newly formed hypoxic tumors.

## 3 The role of mitophagy and ferroptosis in ischemic reperfusion injury of stroke

### 3.1 The role of mitophagy in ischemic-reperfusion injury of stroke

The brain is the most metabolically active organ in the body, with the brain’s oxygen consumption accounting for 20%–30% of the body’s total oxygen consumption and a 24-h glucose consumption of approximately 108 g. The brain’s energy mainly comes from aerobic metabolism of glucose, with almost no energy reserves. Therefore, the brain tissue is highly sensitive to ischemia and hypoxic damage. Studies have shown that the lack of ATP generation caused by ischemia and hypoxia is the main cause of cell and brain tissue death after ischemic stroke (Schädlich et al., 2023). Given the high energy demand of the brain, it is particularly important for mitochondria to produce a large amount of ATP through the respiratory chain and oxidative phosphorylation to ensure mitochondrial morphology and function, and to provide stable and continuous energy supply. Ischemic stroke is a complex series of pathological processes. When ischemic stroke occurs, mitochondria in the ischemic and hypoxic environment have insufficient ATP generation, continuous opening of ion channels causes Ca^2+^ influx, lipase and protease are activated, the mitochondrial respiratory chain is dysfunctional, and a large amount of reactive oxygen species are produced, leading to mitochondrial structural changes and functional impairment (Sims and Muyderman, 2010).

Mitochondria are key organelles that maintain cellular metabolism, regulating the number, morphology, quality, and distribution of mitochondria within cells through activities such as division, fusion, and autophagy. When cerebral ischemia occurs, mitochondrial division and fusion transiently maintain structural integrity and normal function. During ischemic stroke, mitochondria promote the separation of damaged mitochondria through division to maintain mitochondrial health. Studies have shown that significant mitochondrial division can be observed following ischemic stroke and during the ischemia-reperfusion injury period. When ischemic stroke occurs, mitochondrial dysfunction triggers abnormal mitochondrial division and fusion, affecting mitochondrial dynamics and leading to cell death. Optic atrophy 1 (OPA1), as a mitochondrial inner membrane protein, primarily mediates IMM fusion, and research has shown that maintaining the stability of transmembrane long form L-OPA1 located on the inner membrane of mitochondria, so as to reduce neuronal apoptosis and protect ischemic brain tissue. By observing the effect of L-OPA1 on rats with middle cerebral artery occlusion (MCAO) model, it was found that L-OPA1 over-expression could restore mitochondrial morphology and ultrastructure, improve mitochondrial dysfunction, and protect neurons. It is generally believed that mitochondrial fusion can repair mildly damaged mitochondria and promote mitochondrial fusion by up-regulating OPA1 expression levels, thereby providing neuroprotection after ischemia-reperfusion injury. Drp1 is located on the surface of OMM and plays a crucial role in ischemic stroke, and downregulation of Drp1 can inhibit mitochondrial fission and reduce brain injury. As Zhang YH *et al.* (Zhang et al., 2018) observed the effects of crocin on the mitochondrial dynamics of hypoxia-reoxygenation injured human neuroblastoma cells (SH-SY5Y), it was found that crocin can inhibit Drp1 expression and upregulate OPA1 expression, thereby protecting neurons and cells by suppressing mitochondrial division and fusion abnormalities. Other studies have shown that ginkgolide K has a significant inhibitory effect on Drp1, and in the MCAO model, ginkgolide K can alleviate neuronal damage by preventing GSK3β and Drp1 translocation to mitochondria (Zhou et al., 2017). Additionally, Grohm *et al.* (Grohm et al., 2010) found that mitochondrial fusion could repair mild damage and decrease the infarct size, indicating that mitochondrial dynamics played an important role in the ischemic stroke. Inhibiting mitochondrial division or promoting fusion may offer new therapeutic targets for treating such diseases. It is noteworthy that following ischemic stroke, the accumulation of ROS within cells can cause mitochondrial depolarization, thereby initiating autophagy. Increasing research suggests that mitochondrial autophagy is closely associated with ischemic stroke, cardiovascular disease, and neurodegenerative disorders (Shao et al., 2020; Ajoolabady et al., 2022; Li et al., 2023b). Some studies related to stroke suggest that mitochondrial autophagy appears to be a cell survival mechanism because its ability to engulf and clear damaged mitochondriafacilitates the maintenance of organelle integrity and cellular energy supply, reduces cellular and neuronal death, and thus serves as a neuroprotective agent to ameliorate clinical symptoms after stroke. Research has confirmed that in stroke models, BNIP3 and NIX interact, and downregulating BNIP3 leads to weakened mitochondrial autophagy, while NIX activation shows similar changes. As Yuan Y *et al.* (Yuan et al., 2017) discovered, in ischemia-reperfusion models, knocking out NIX decreased mitochondrial autophagy, exacerbated brain damage, indicating that NIX had neuroprotective effects and suggesting that mitochondrial autophagy mediated by BNIP3/NIX might be a potential therapeutic target for ischemic stroke. However, mitochondrial autophagy is not always protective, excessive mitochondrial autophagy can lead to cell death. For example, Zhang YF and others (Zhang et al., 2020) found that peroxynitrite (ONOO) mediated Drp1 recruitment to damaged mitochondria, causing excessive mitochondrial autophagy and exacerbating cerebral ischemia-reperfusion injury, indicating that ONOO-mediated mitochondrial autophagy activation might be a key therapeutic target for improving ischemic stroke prognosis. Thus, mitochondrial autophagy is a double-edged sword. On one hand, it can maintain cellular homeostasis by clearing damaged mitochondria. On the other hand, excessive autophagy can lead to excessive mitochondrial degradation.

In conclusion, the interplay between mitochondrial dynamics and mitochondrial autophagy plays a crucial role in the ischemic stroke. Mitochondria not only produce ATP, but also serve as an important source of ROS, which is a determining factor in the degree of ischemia-reperfusion injury. Although ischemic stroke leads to mitochondrial dysfunction and destructive effects, when reperfusion occurs, the rapid restoration of oxygen levels causes excessive activation of the mitochondrial electron transport chain, leading to increased levels of ROS and exacerbating cerebral ischemic damage. Therefore, prolonging the reperfusion window and providing neuroprotection are crucial for disease treatment. Furthermore, mitochondrial autophagy serves as an early defensive mechanism in ischemia-reperfusion injury, clearing damaged mitochondria and reducing the stimulation and damage to normal mitochondria. However, when autophagy is excessive or blocked, it may exacerbate the damage. Therefore, reasonably regulating the mitochondrial autophagy flux during different stages of ischemic stroke is essential for disease treatment and prognosis.

### 3.2 The role of ferroptosis in ischemia-reperfusion injury of stroke

During ischemic brain tissue, increased ROS, weakened antioxidant defense, excessive free radical production, inactivation of antioxidant enzymes, and consumption of antioxidants lead to various harmful events, including lipid and protein peroxidation, DNA damage, and cell death. Multiple studies have shown that ferroptosis plays a regulatory role in ischemic brain damage (mainly in neurons) and mediates the occurrence and development of rat ischemic brain injury, with inhibition of ferroptosis reducing rat ischemic brain damage. As Tuo QZ *et al.* (Tuo et al., 2017) discovered, increased iron load after cerebral ischemia exacerbates cell death in brain tissue, and administration of ferroptosis inhibitors such as Ferrostatin-1 (Fer-1) and Liproxstatin-1 (Lip-1) protected mice from ischemia-reperfusion injury caused by middle cerebral artery occlusion, confirming that ferroptosis can lead to neuron death after ischemic brain injury. Interestingly, further findings suggested that tau gene knockout mice were protected from ferroptosis after ischemia-reperfusion injury, suggesting that tau-iron interaction may be one of the mechanisms underlying ferroptosis. In a rat middle cerebral artery occlusion model, increased iron concentration in the damaged brain region may be caused by vascular leakage nearby, leading to the influx of transferrin, which increases the saturation of plasma transferrin and exhibits neuroprotective effects. Lan B *et al.* (Lan et al., 2020) found that the expression of divalent metal ion transporter 1 (DMT1) was upregulated in the rat middle cerebral artery occlusion model, and DMT1 inhibition suppressed ferroptosis and alleviated cerebral ischemic damage. During the ischemic period, the expression of brain transferrin receptor 1 (TFR1) increased, and extracts from the traditional Chinese medicine Naotai formula can reduce TFR1 expression to inhibit ferroptosis and improve brain damage. GPX4 and GSH are endogenous ferroptosis inhibitors that are closely related to cerebral ischemia. In ischemic rats, low GPX4 levels lead to ferroptosis, while increased GPX4 levels can improve cerebral damage. Phellandrene is a drug used to treat cerebral ischemia, and it can inhibit ferroptosis by enhancing GPX4 expression and reducing hippocampal neuron damage in the process of cerebral ischemia-reperfusion injury. Lipoxygenases (LOXs) are key enzymes that oxidize polyunsaturated fatty acids (PUFAs) to cause lipid peroxidation and ferroptosis. They are highly expressed after cerebral ischemia and can be inhibited to alleviate damage. There are several subtypes of LOXs, including 12/15-LOX. In the middle cerebral artery occlusion model, 12/15-LOX expression increased, and inhibition of 12/15-LOX can reduce neuron death, thus improving neuron recovery.

In summary, cerebral ischemia can induce ferroptosis, which in turn exacerbates cerebral ischemic damage. Inhibition of ferroptosis can alleviate this ischemic damage. However, the regulatory mechanisms involving brain ferroptosis remain unclear, including the upstream regulatory mechanisms of iron overload, GPX4, and 12/15-LOX. It is still uncertain whether ferroptosis directly causes cell death or indirectly causes it through other mechanisms, which requires further exploration by scholars.

### 3.3 The role of crosstalk between mitophagy and ferroptosis in ischemia-reperfusion injury of stroke

Reperfusion injury after cerebral ischemia is a complex pathological process caused by the restoration of blood supply following ischemia, involving various mechanisms such as oxidative stress, inflammatory responses, cellular apoptosis, and so on. Recently, it has been found that mitophagy and ferroptosis are also two closely related modes of cell death associated with cerebral ischemia-reperfusion injury. Currently, research on the crosstalk mechanisms between them is gradually unfolding. However, this research field is relatively new, with continuous new discoveries being made.

Mitophagy is a specific autophagic process that primarily functions to maintain mitochondrial quality and cellular homeostasis by eliminating damaged mitochondria. On the other hand, ferroptosis is a non-apoptotic form of cell death that depends on iron, characterized by the accumulation of lipid peroxides and inactivation of antioxidant repair systems. Some studies have suggested that in a model of ischemia-reperfusion injury in stroke, the activity of mitophagy may be regulated and interact with the iron metabolism and ferroptosis signaling pathways. As Lin QS *et al.* (Lin et al., 2023) found that mitophagy could regulate iron ion levels through the ROS/HO-1/GPX4 axis, thereby alleviating cisplatin-induced ferroptosis in renal tubular epithelial cells. Another study indicated that the activity of mitophagy may be inhibited during the progression of ischemia-reperfusion injury in stroke, leading to excessive accumulation of iron ions in cells and subsequently inducing ferroptosis (Tian et al., 2023). On the other hand, the dysregulation of iron metabolism and the occurrence of ferroptosis also control the autophagic flux of mitochondria in the ischemia-reperfusion injury model of stroke. According to research, mitophagy may lead to an increase in free iron content within neurons by degrading ferritin, thereby promoting iron-dependent neuronal death (Ajoolabady et al., 2021; Bi et al., 2021). The main mechanism appears to involve the inhibition of JNK protein phosphorylation by FUNDC1 protein (Wu et al., 2019b), which is believed to suppress the transcription of Nuclear receptor coactivator-4 (NCOA4) (Guo et al., 2021a), leading to increased degradation of cytoplasmic ferritin and release of free Fe^2+^, thereby inducing iron-dependent cell death (Peng et al., 2016). In summary, there is an active crosstalk between mitophagy and ferroptosis in ischemia-reperfusion injury in stroke, mainly manifested in three aspects: (1) Damaged mitochondria may release a large amount of iron ions into the cytoplasm, triggering iron-dependent lipid peroxidation and promoting ferroptosis. (2) Levels of ROS are significantly increased in ischemia-reperfusion injury in stroke. ROS can not only directly damage mitochondria, leading to mitophagy, but also promote lipid peroxidation reactions and enhance ferroptosis. Therefore, ROS may be an important bridge linking mitophagy and ferroptosis. (3) Mitophagy and ferroptosis may mutually regulate each other through sharing certain signaling pathways. For example, nuclear factor E2-related factor 2 (Nrf2) is not only a key regulator of the antioxidant stress response, but also a modulator of iron metabolism. Its activation can simultaneously affect mitophagy and ferroptosis. Further research can focus on the role and mechanism of Nrf2 in regulating these two modes of cell death.

In conclusion, there is a complex crosstalk regulation mechanism between mitophagy and ferroptosis in cerebral ischemia-reperfusion injury, and the modulation of mitophagic activity may affect iron metabolism and the occurrence of ferroptosis, while abnormal levels of iron ions also affect the activity of mitophagy. Therefore, further research will help to reveal this crosstalk of regulatory mechanism, providing new targets and strategies for the treatment of ischemia-reperfusion injury in stroke.

## 4 Medicinal plants and their active metabolites alleviate cerebral ischemia-reperfusion injury by targeting mitophagy and ferroptosis

In recent years, an increasing number of researchers have pointed out that medicinal plants have the characteristics of “multi-component, multi-target, and multi-pathway,” which is precisely in line with the complexity and variability of the evolution process of cerebral stroke and its complications. Moreover, according to modern pharmacological research, medicinal plants and their active metabolites not only have extensive biological activities (such as antioxidant, anti-inflammatory, and anti-tumor), but they have also been proven to be relatively safe, especially compared to western drugs in terms of liver and kidney toxicity damage. Therefore, the research and development of medicinal plants (including single-medicinal plant and traditional Chinese herbal compound preparations) and their active metabolites are very promising (Tables 1–6), which greatly brings hope for patients with cerebral stroke and its complications.

**TABLE 1:** Traditional Chinese herbal compound preparations alleviate the degree of cerebral ischemia-reperfusion injury by regulating mitophagic flux.

Chinese herbal compound preparations	Medicinal plants	Autophagy-related targets	*In vitro*/*In vivo*	Model	References
Taohong Siwu decoction	*Bupleurum chinense DC., Angelica sinensis (Oliv.) Diels*	LC3B	*In vivo*	MCAO/R-induced SD rat model	Shi et al. (2023)
	*Paeonia lactiflora Pall., Conioselinum anthriscoides “Chuanxiong”, Salvia miltiorrhiza Bunge*				
	*Smilax glabra Roxb., Plantago asiatica L., Buddleja officinalis Maxim.*				
	*Tribulus terrestris L., Alisma plantago-aquatica L., Leonurus japonicus Houtt.,*				
	*Senna tora (L.) Roxb*				
Xiao-Xu-Ming decoction	*Ephedra sinica* Stapf	LC3, Beclin1, Lamp1, SQSTM1/p62	*In vivo*	MCAO/R-induced SD rat model	Lan et al. (2018)
	*Stephania tetrandra S.Moore, Panax ginseng C.A.Mey*				
	*Scutellaria baicalensis Georgi*				
	*Neolitsea cassia (L.) Kosterm*				
	*Glycyrrhiza glabra L.*				
	*Paeonia lactiflora Pall., Conioselinum anthriscoides “Chuanxiong”*				
	*Juglans regia L.*				
	*Aconitum carmichaelii Debeaux, Saposhnikovia divaricata (Turcz.) Schischk., Zingiber officinale Roscoe*				
Longhu Xingnao granules	*Pheretima, Ambrum, Astragalus membranaceus (Fisch.) Bunge, Angelica sinensis (Oliv.) Diels, Conioselinum anthriscoides “Chuanxiong,” Juglans regia L., Paeonia lactiflora Pall., Gastrodia elata Blume, Typha domingensis Pers., Rheum officinale Baill., Panax bipinnatifidus Seem., Ziziphus jujuba Mill*	LC3B, Beclin-1, BNIP-3	*In vivo*	MCAO/R-induced SD rat model	Zhang et al. (2022b)
Huoxue Rongluo decoction	*Spatholobus suberectus Dunn*	LC3B, Pink1, Parkin, SQSTM1/p62, TOMM20	*In vitro*	OGD/R-induced PC12 cell model	Yan et al. (2023)
	*Pourthiaea villosa (Thunb.) Decne., Rehmannia glutinosa (Gaertn.) DC., Curcuma aeruginosa Roxb., Polygonatum cyrtonema Hua*				
	*Boswellia sacra Flück., Commiphora myrrha (T.Nees) Engl., Conioselinum anthriscoides “Chuanxiong”*				
Huazhuo Jiedu Huoxue Tongluo decoction	*Coptis chinensis Franch.*	Pink1, Parkin	*In vivo*	MCAO/R-induced SD rat model	Sun et al. (2023)
	*Smilax glabra Roxb., Alisma plantago-aquatica L.,*				
	*Salvia miltiorrhiza Bunge, Paeonia lactiflora Pall., Angelica sinensis (Oliv.) Diels, Dianthus chinensis L., Curcuma aromatica Salisb., Conioselinum anthriscoides “Chuanxiong,” Pheretima, Glycyrrhiza glabra L*				
Qizhi granules	*Astragalus membranaceus (Fisch.) Bunge, Reynoutria multiflora (Thunb.) Moldenke, Whitmania pigra Whitman, Juglans regia L., Curcuma phaeocaulis Valeton, Sparganium stoloni erum, Buch. -Ham., Senna tora (L.) Roxb.,*	LC3B, SQSTM1/p62	*In vivo*	MCAO/R-induced SD rat model	Duan et al. (2023)
	*Crataegus pinnatifida Bunge, Trionyx sinensis Wiegmann*				

**TABLE 2:** Traditional Chinese herbal compound preparations alleviate the degree of cerebral ischemia-reperfusion injury by regulating ferroptosis levels.

Chinese herbal compound preparations	Medicinal plants	Ferroptisis-related targets	*In vitro*/*In vivo*	Model	References
Xingnaojing injection	*Moschus, Curcuma aromatica Salisb., Gardenia jasminoides J. Ellis, Dryobalanops aromatica C.F.Gaertn*	GPX4, FPN, TFR, DMT1	*In vitro* *,* *in vivo*	Hypoxia SH-SY5Y cell model, MCAO/R-induced SD rat model	Liu et al. (2023a)
Naotai formula	*Pheretima, Astragalus membranaceus (Fisch.) Bunge, Ligusticum sinense Oliv., Bombyx mori Linnaeus*	BMP6, SMADs, FPN, SLC40A1, GSH, GPX4	*In vitro*	OGD/R-induced BV2 cell model	Liao et al. (2023)
Compound tongluo decoction	*Pleuropterus multiflorus (Thunb.) Nakai, Polygonatum cyrtonema Hua, Ecklonia kurome, Bombyx mori Linnaeus, Euonymus alatus (Thunb.) Sieb, Gastrodia elata Blume, Whitmania pigra Whitman*	SHH, Gli1, GPX4, ACSL4, ALOX5	*In vitro* *,* *in vivo*	OGD/R-induced hippocampal neurons cell model, MACO/R-induced rat model	Hui et al. (2022)
Wenyang Fuyuan decoction	*White appendage, Astragalus membranaceus (Fisch.) Bunge, Codonopsis pilosula (Franch.) Nannf., Dianthus chinensis L., Epimedium brevicornu Maxim., Panax notoginseng (Burkill) F. H. Chen ex C. H. Chow, Zingiber oj-jicinale Rosc., Glycyrrhiza glabra L*	TFR1, GSH, IRP1, FPN	*In vivo*	MCAO/R-induced SD rat model	Li et al. (2023)
Buyang Huanwu decoction	*Astragalus membranaceus (Fisch.) Bunge, Angelica sinensis (Oliv.) Diels, Paeonia lactiflora Pall., Pheretima, Conioselinum anthriscoides “Chuanxiong,” Juglans regia L., Carthamus tinctorius L*	TfR1, DMT1, FPN1, GPX4	*In vitro* *,* *in vivo*	OGD/R-induced HT22 cell model, MCAO/R-induced SD rat model	Cai (2023)

**TABLE 3:** A single medicinal plant alleviates the degree of cerebral ischemia-reperfusion injury by modulating mitophagic flux.

Single medicinal plants	Autophagy-related targets	*In vitro*/*In vivo*	Model	References
*Dengzhan Xixin injection*	LC3B, SQSTM1/p62, TOM20, Pink1, Parkin	*In vivo*	MCAO/R-induced SD rat model	Yang et al. (2022)
*Saffron extract*	LC3B, AMPK, mTOR	*In vitro*	OGD/R induced HT22 cell model	Huang et al. (2019)
*Ginkgo biloba extract*	Beclin1, LC3B, SQSTM1/p62	*In vivo*	MCAO/R-induced SD rat model	Zang et al. (2023)

**TABLE 4:** A single medicinal plant alleviates the degree of cerebral ischemia-reperfusion injury by modulating ferroptosis levels.

Single medicinal plants	Autophagy-related targets	*In vitro*/*In vivo*	Model	References
*Paeoniae Radix Rubra*	GPX4, FTH1	*In vitro* *,* *in vivo*	HT22 cell line following oxidative stress model, MCAO injury SD rat model	Zhao et al. (2023a)
*Neutral polysaccharide from Gastrodia elata*	GPX4	*In vitro* *,* *in vivo*	OGD/R induced HT22 cell model	Zhang et al. (2023)
			IS mouse model	
*Roots of Astragalus*	Fn, FHC, FLC, Tf, TfR, DMT1, LTCC, TRPC6, FPN1, SLC3A2, GPX4	*In vivo*	MCAO/R-induced SD rat model	Chen et al. (2022)

**TABLE 5:** The active metabolite alleviates the degree of cerebral ischemia-reperfusion injury by modulating mitophagic flux.

Metabolites	Medicinal plants	Autophagy-related targets	*In vitro*/*In vivo*	Model	References
Ligustilide	*Conioselinum anthriscoides “Chuanxiong” and Angelica sinensis (Oliv.) Diels*	Parkin, Pink1	*In vitro* *,* *in vivo*	OGD/R-induced hippocampal neurons cell model, MCAO/R-induced SD rat model	Mao et al. (2022)
Artemisinin	*Artemisia Annua L*	PHB2, Tomm20, SQSTM1/p62, LC3B	*In vitro*	OGD/R-induced SH-SY5Y cell model	Jiang et al. (2022)
Jionoside A1	*Rehmannia glutinosa (Gaertn.) DC.*	LC3B, SQSTM1/p62, Nix, Parkin, FUNCD1	*In vitro* *,* *in vivo*	OGD/R-induced cell model, tMCAO/R-induced SD rat model	Yu et al. (2023)
Ginsenoside compound K	*Panax ginseng C.A.Mey*	LC3B, Tomm20	*In vitro* *,* *in vivo*	OGD/R-induced PC12 cell model, MCAO/R-induced SD rat model	Huang et al. (2023)
Panax notoginseng saponins	*Panax Notoginseng (Burk.) F. H. Chen Ex C. Chow*	Parkin, Pink1	*In vivo*	MCAO/R-induced SD rat model	Xiao et al. (2022)
Curcumin	*Curcuma longa L.*	LC3B	*In vitro* *,* *in vivo*	OGD/R-induced neurons cell model, MCAO/R-induced SD rat model	Wang and Xu (2020)
Naringin	*Citrus reticulata Blanco*	LC3B, Parkin	*In vitro* *,* *in vivo*	OGD/R-induced SH-SY5Y cell model, MCAO/R-induced SD rat model	Feng et al. (2018)
Garciesculenxanthone B	*Garcinia hanburyi Hook.f.*	Parkin, Pink1, LC3B,SQSTM1/p62, Tomm20, Timm23, MFN1	*In vitro* *,* *in vivo*	OGD/R-induced HeLa and SH-SY5Y cell model, MCAO/R-induced mice model	Wu et al. (2021a)
Salidroside	*Rhodiola rosea L*	Parkin, Pink1, LC3B, p62, Tomm20	*In vitro* *,* *in vivo*	OGD/R-induced neuronal cell model, MCAO/R-induced C57BL/6 mice model	Gu et al. (2020)
Esculetin	*Aesculus chinensis Bunge*	Bnip3, Beclin1, Pink1, Parkin, LC3B	*In vivo*	tMCAO/R-induced mice model	Xu et al. (2019)
Baicalin	*Scutellaria baicalensis Georgi*	Drp1, AMPK, SQSTM1/p62	*In vitro* *,* *in vivo*	OGD/R-induced PC12 cell model, MCAO/R-induced SD rat model	Li et al. (2017)
Resveratrol	*Polygoni Cuspidati Rhizoma Et Radix*	LC3B, Timm23, Tomm20, Pink1, Parkin	*In vitro*	OGD/R-induced primary cortical neurons cell model	Ye et al. (2021)
Oridonin	*Isodon rubescens (Hemsl.) H.Hara*	RIPK3, AMPK, Pink1, Parkin, LC3B, SQSTM1/p62	*In vivo*	tMCAO/R-induced mice model	Li et al. (2023c)
Rehmapicroside	*Rehmannia glutinosa (Gaertn.) DC.*	Parkin, Pink1, SQSTM1/p62, LC3B	*In vitro* *,* *in vivo*	OGD/R-induced PC12 cell model, MCAO/R-induced SD rat model	Zhang et al. (2020)

**TABLE 6:** The active metabolite alleviates the degree of cerebral ischemia-reperfusion injury by modulating ferroptosis levels.

Metabolites	Medicinal plants	Ferroptosis-related targets	*In vitro*/*In vivo*	Model	References
*β*-Caryophyllene	*Dianthus chinensis L*	ACSL4, COX2, GPX4	*In vitro* *,* *in vivo*	OGD/R-induced primary astrocytes model, MCAO/R-induced SD rat model	Hu et al. (2022)
Carthamin yellow	*Carthamus tinctorius L*	ACSL4, FTH1, GPX4, TFR1	*In vivo*	MCAO/R-induced SD rat model	Guo et al. (2021b)
Baicalein	*Scutellaria baicalensis Georgi*	GPX4, ACSL4, ACSL3	*In vitro* *,* *in vivo*	OGD/R-induced HT22 cell model, tMCAO/R-induced mice model	Li et al. (2022a)
Rehmannioside A	*Rehmannia glutinosa (Gaertn.) DC.*	SLC7A11, GPX4	*In vitro* *,* *in vivo*	H2O2-induced SH-SY5Y cell model, MCAO/R-induced SD rat model	Fu et al. (2022)
Dihydromyricetin	*Nekemias megalophylla (Diels and Gilg) J.Wen and Z.L.Nie*	GPX4, ACSL4, PEBP1	*In vitro* *,* *in vivo*	OGD/R-induced HT22 cell model, MCAO/R-induced SD rat model	Xie et al. (2022)
Astragaloside IV	*Hedysarum Multijugum Maxim*	GPX4, GSH	*In vitro* *,* *in vivo*	OGD/R-induced SH-SY5Y cell model, MCAO/R-induced SD rat model	Wang et al. (2023a)
Kaempferol	*Kaempferiae Rhizoma*	SLC7A11, GPX4	*In vitro*	OGD/R-induced neuronal injury	Yuan et al. (2021)
Procyanidins	*Vitis vinifera L*	GPX4, SLC7A11	*In vivo*	MCAO/R-induced mice model	Chen et al. (2023)
Galangin	*Alpiniae Officinarum Rhizome*	SLC7A11, GPX4	*In vivo*	Using bilateral common carotid artery ligation in gerbils	Guan et al. (2021)
Vitexin	*Viticis Negundo Folium*	Tfr1, SLC7A11, GPX4	*In vitro* *,* *in vivo*	OGD/R-induced neuron cell model, MCAO/R-induced SD rat model	Guo and Shi (2023)
Oxysophoridine	*Sophora flavescens Aiton*	ACSL4, TFR1, FTH1, GPX4	*In vitro* *,* *in vivo*	OGD/R-induced HT22 cell model, MCAO/R-induced SD rat model	Zhao et al. (2023b)
Ginkgolide B	*Ginkgo biloba L*	ACSL4, GPX4, FTH1, NCOA4	*In vitro* *,* *in vivo*	OGD/R-induced PC12 cell model, tMCAO/R-induced SD rat model	Yang et al. (2024)
Resveratrol	*Oroxylum indicum (L.) Kurz*	SLC7A11, TFR1, GPX4, ACSL4, PTGS2	*In vivo*	MCAO/R-induced SD rat model	Li et al. (2022b)
Berberine	*Coptis chinensis Franch*	GPX1	*In vivo*	MCAO/R-induced mice model	Wang et al. (2023b)
Soybean isoflavones	*Glycine max (L.) Merr*	GPX4	*In vivo*	MCAO/R-induced SD rat model	Li et al. (2023d)

Abbreviations: MCAO/R, middle cerebral artery occlusion/reperfusion; SD, sprague dawley; OGD/R, oxygen-glucose deprivation/reoxygenation; tMCAO/R, transient middle cerebral artery occlusion/reperfusion.

### 4.1 Traditional Chinese herbal compound preparations alleviate cerebral ischemia-reperfusion injury by regulating mitophagy

Taohong Siwu decoction (THSWD) has the efficacy of promoting blood circulation and removing blood stasis and is a commonly used traditional Chinese medicine compound formulation for treating ischemic stroke. It is composed of *Paeonia lactiflora Pall.*, *Conioselinum anthriscoides (H.Boissieu) Pimenov* and *Kljuykov*, *Rehmannia glutinosa (Gaertn.)*. The ratio of *DC.*, *Prunus persica (L.) Batsch*, *Angelica sinensis (Oliv.) Diels*, and *Carthamus creticus L.* is 3:2:4:3:3:2. Notably, Shi Y *et al.* (Shi et al., 2023) studied the protective effects and mechanisms of THSWD on PC12 cells injured by oxygen-glucose deprivation/reperfusion (OGD/R) *in vitro*. They used the PC12 cell OGD/R model to simulate *in vitro* neuron ischemia-reperfusion injury, and evaluated the severity of PC8 cell damage by CCK-8, flow cytometry, and lactate dehydrogenase (LDH) assays. They also observed the ultrastructure of mitochondria and ferroptosis by transmission electron microscopy, and assessed mitochondrial function using ATP and mitochondrial membrane potential (MMP) detection kits. Additionally, they detected cell autophagy and NLRP3 inflammasome-related proteins by western blot and immunofluorescence staining. The results showed that THSWD treatment improved the survival rate of OGD/R-damaged PC12 cells, reduced cell damage and apoptosis. Moreover, the expression of ATP, MMP, and autophagy markers (LC3-II/LC3-I, Beclin1, Atg5) and mitochondrial autophagy markers (Parkin and PINK-1) was significantly increased, while the levels of ROS, NLRP3 inflammasome, and pro-inflammatory cytokines were significantly decreased. These beneficial effects of THSWD on mitochondrial autophagy and NLRP3 inflammasome were reversed by mitochondrial division inhibitory factor 1 (Mdivi-1). The study indicates that THSWD can protect PC3 cells from OGD/R damage by enhancing mitochondrial autophagy and inhibiting the activation of NLRP12 inflammasome, which provides a certain theoretical basis for the application of THSWD in ischemic stroke and reperfusion injury.

Xiao-Xu-Ming decoction (XXMD), with the efficacy of warming the Yang, promoting blood circulation, and removing blood stasis, has been widely used in the treatment of ischemic stroke, and its clinical effect is significant. It is composed of herbs such as ephedra, aristolochia, panax ginseng, scutellaria baicalensis, cinnamon, licorice, peony, ligusticum chuanxiong, apricot kernel, aconitum, radix saposhnikoviae, and ginger. Previous studies have shown that XXMD can protect neurons, blood vessels, and mitochondria from cerebral ischemia-reperfusion-induced damage (Zhu et al., 2010; Lan et al., 2013, 2014). However, it remains unclear whether XXMD can regulate mitochondrial autophagy after cerebral ischemia-reperfusion. Therefore, Lan R *et al.* (Lan et al., 2018) investigated the effects of XXMD on mitochondrial autophagy and mitochondrial function after cerebral ischemia-reperfusion. They used triphenyltetrazolium chloride (TTC) staining to measure the infarct area, hematoxylin and eosin (HE) staining and Nissl staining to assess cerebral ischemic damage, and transmission electron microscopy to observe the ultrastructural characteristics of mitochondria and mitochondrial autophagy in ischemic cerebral cortex. Subsequently, they also detected mitochondrial autophagy by immunofluorescence labeled with LC3B and VDAC1, observed the formation of autophagosomes labeled with LC3B and Lamp1, and analyzed the expression levels of LC3B, Beclin1, and Lamp1 proteins by western blot. The results showed that the neurological score worsened and cell ischemic damage was severe in MCAO rats. However, these phenomena were significantly reversed by XXMD. In addition, XXMD significantly downregulated mitochondrial autophagy and reduced the expression levels of LC3B, Beclin1, and Lamp1 proteins induced by cerebral ischemia-reperfusion. This study suggests that XXMD exerts neuroprotective effects by down-regulating the expression levels of LC3B, Beclin1, and Lamp1 proteins, thereby reducing mitochondrial activation and improving mitochondrial function in cerebral ischemia-reperfusion injury.

### 4.2 Traditional Chinese herbal compound preparations alleviate cerebral ischemia-reperfusion injury by regulating ferroptosis

Xingnaojing injection (XNJ) is made of natural musk, gold, gardenia, borneol and other herbs through scientific methods, which has the effect of clearing heat, cooling blood, and promoting blood circulation. XNJ is a neuroprotective traditional Chinese medicine injection, which has been widely used in the treatment of stroke. Liu HQ *et al.* (Liu et al., 2023a) explored the potential mechanism of XNJ in ferroptosis-mediated cerebral ischemia using proteomics and *in vitro* and *in vivo* experiments. They found that XNJ treatment reduced the infarct volume and brain tissue damage in MCAO rats. Nissl staining also showed that compared with MCAO rats, the brain tissue Nissl bodies in XNJ-treated rats were clear, increased by 3.54 times, indicating that XNJ improved the cerebral damage and neurological deficit in MCAO rats. Proteomics identified 101 differentially expressed proteins (DEPs) shared by both conditions. Interestingly, these DEPs were closely related to ferroptosis according to bioinformatics analysis. Further studies showed that after XNJ treatment, the expressions of GPX4, ferroportin (FPN), and heme oxygenase-1 (HO-1) were upregulated, while the expressions of cyclooxygenase-2 (COX-2), transfer receptors (TFR), and divalent metal transporter-1 (DMT1) were downregulated, which relieved MCAO-induced cerebral ischemia. In addition, *in vitro* experiments showed that XNJ increased the survival rate of hypoxic injured SH-SY5Y cells. XNJ increased the level of GPX4 after cell hypoxia, inhibited the protein expression of COX-2 and TFR. Additionally, XNJ of different concentrations (0.25%, 0.5%, 1%) decreased the ROS content in hypoxic cells, indicating that XNJ can inhibit hypoxia-induced cell damage by regulating the expression of ferroptosis-related proteins and reducing ROS production. This study suggests that XNJ can promote the recovery of neurological function in MCAO rats and hypoxic SH-SY5Y cells by regulating ferroptosis.

Naotai formula (NTF) is a novel traditional Chinese medicine therapy for ischemic stroke, showing beneficial effects in inhibiting inflammation and lipid peroxidation synthesis. Liao J *et al.* (Liao et al., 2023) established an OGD/R model using BV2 microglial cells and detected the effects of NTF on inflammation and ferroptosis in OGD/R-damaged BV2 microglial cells using immunofluorescence, fluorescence probe method, DCFH-DA flow cytometry, enzyme-linked immunosorbent assay (ELISA), and western blot technology. The results showed that microglial M1 polarization promotes the secretion of pro-inflammatory cytokines and exacerbates ferroptosis and brain damage after OGD/R surgery. However, the BMP6 inhibitor LND-193189 reversed these effects. Similarly, NTF promotes the shift of microglia from M1 to M2. In addition, NTF effectively inhibits the expression of ferromodulin, BMP6 and SMADs, and promotes the expression levels of ferroportin (FPN, SLC40A1) and GPX4. This study indicates that microglial M1/M2 polarization plays a crucial role in inflammation and ferroptosis during OGD/R. The BMP6/SMADs signaling pathway is a potential therapeutic target for microglial transformation-induced inflammation and ferroptosis. Notably, NTF can alleviate inflammation and ferroptosis in BV2 microglial cells by regulating the BMP6/SMADs signaling pathway in OGD/R-induced damage.

Compound tongluo decoction (CTLD) is a traditional Chinese medicine formula with various pharmacological activities, including improving cerebral ischemia symptoms. To further explore the potential mechanism of CTLD in relieving cerebral ischemia, Hui Z *et al.* (Hui et al., 2022) established MCAO rat models and OGD/R cell models for research. The expression of endoplasmic reticulum (ER) stress, ferroptosis, Sonic Hedgehog (SHH) pathway-related proteins, and angiogenesis-related proteins were analyzed by Western blot. The expression of CD31 was detected by immunofluorescence to study angiogenesis. Furthermore, immunohistochemistry was used to detect the expression of GRP78 and XBP-1 in brain tissue. Prussian blue staining was applied to detect iron deposition in brain tissue. ELISA kits were used to measure ROS, malondialdehyde (MDA), and superoxide dismutase (SOD) levels. Angiogenesis was analyzed by tube formation assays. The results showed that CTLD and 4-phenylbutyric acid (4-PBA, a ER stress inhibitor) can alleviate cerebral ischemia symptoms. At the molecular level, CTLD and 4-PBA rescued ER stress and ferroptosis but promoted the SHH signaling pathway in cerebral infarction rats. Interestingly, cerebral infarction showed high levels of angiogenesis, and CTLD exacerbated angiogenesis but 4-PBA inhibited it. In addition, CTLD inhibited ER stress and ferroptosis but promoted angiogenesis in OGD/R-induced PC12 cells, which was partially reversed by the SHH signaling antagonist SANT-1. Overall, this study revealed that CTLD may alleviate cerebral ischemia symptoms by activating the SHH pathway in cerebral infarction rats, inhibiting ER stress-induced ferroptosis, and promoting angiogenesis.

### 4.3 Single-medicinal plant alleviate cerebral ischemia-reperfusion injury by regulating mitochondrial autophagy

Dengzhan Xixin injection (DX) is an herbal extract derived from the medicinal plant *Brachypodium distachyon*, which has been widely used in the clinical treatment of sequelae of cerebral ischemia. Lv Y *et al.* (Lv et al., 2022) detected the main components of DX by high-performance liquid chromatography (HPLC) and established an SD rat brain ischemia-reperfusion injury model using MCAO. They found that DX mainly contains baicalein, 3,4-O-dicaffeoylquinic acid, 3,5-O-dicaffeoylquinic acid, 4,5-O-dicaffeoylquinic acid, caffeic acid, and 5-O-caffeoylquinic acid. Compared with the model group, DX significantly alleviated neurological deficits, reduced rCBF deficiency, and cerebral infarction symptoms. Furthermore, the pathological changes and neuron loss in the rat MCAO model were significantly improved after DX administration. Simultaneously, DX decreased the increased levels of ROS and MDA while increasing the level of SOD. Notably, DX treatment led to the collapse of ATP and the MMP system, as well as a decrease in the relative copy number of mitochondrial DNA (mtDNA). Increasing the number of autophagosomes helped maintain the ultrastructure of mitochondria. The representative components of DX had potential binding sites for mitochondrial autophagy/apoptosis-related proteins. DX increased the protein expression of LC3B, PINK1, and Parkin while reducing the levels of p62 and TOM20. In addition, DX limited the TUNEL-positive cell rate, decreased the expression of Bax, Cyto-c, and cleaved Caspase-3, and increased the level of Bcl-2. This study confirmed that the protection of DX against cerebral ischemia is attributed to up-regulating mitochondrial autophagy and inhibiting mitochondria-mediated cell apoptosis, thus effectively improving rat cerebral ischemia-reperfusion injury.

### 4.4 Single-medicinal plant alleviate cerebral ischemia-reperfusion injury by regulating ferroptosis

*Paeonia lactiflora*, a natural medicinal plant, has been widely used in clinical practice in China to promote blood circulation and eliminate blood stasis. However, its effects on cerebral ischemia are rarely reported. To evaluate the potential therapeutic effects of *P. lactiflora* extracts on cerebral ischemia and explore its potential mechanisms, Zhao FY *et al.* (Zhao et al., 2023a) conducted a study to preliminarily screen the active ingredients and provide an experimental basis for the potential application of *P. lactiflora* as a novel therapeutic drug. They found that *P. lactiflora* extracts can reduce the infarct volume and improve the neurological deficit in rats, as well as upregulate the expression of GPX4, FTH1, Beclin1, LC3 II, and p-Akt in the hippocampus. Meanwhile, *in vitro* studies showed that *P. lactiflora* extracts could alleviate H_2_O_2_-induced HT22 cell damage by regulating cell factors, such as MDA, GSH, and ROS, accompanied by increased protein expression of GPX4 and Beclin1. Notably, the PI3K/Akt signaling pathway can be inhibited by the PI3K inhibitor LY294002. Further studies confirmed that the effective components of *P. lactiflora* extracts in regulating ferroptosis and autophagy are mainly defined as albiflorin, paeoniflorin, benzoylpaeoniflorin, oleanolic acid, and hesperetin. These data suggest that *P. lactiflora* extracts exert neuroprotective effects by inhibiting ferroptosis and activating autophagy through the PI3K/Akt signaling pathway. This study not only lays a theoretical foundation for the potential application of *P. lactiflora* extracts as a novel therapeutic drug but also provides insights into the PI3K/Akt-related ferroptosis and autophagy as therapeutic targets for cerebral ischemia.

The crosstalk between ferroptosis and neuroinflammation plays a significant role in the pathogenesis of cerebral ischemia-reperfusion injury. Studies have shown that neutral polysaccharides (NPGE) from medicinal plant *Gastrodia elata* have significant effects on oxidative stress and inflammation (Zhu et al., 2019). To investigate the potential impact of NPGE on neuropathology in ischemic brain reperfusion injury, Zhang YG *et al.* (Zhang et al., 2023) conducted a series of experiments by using a mouse model of ischemic stroke and HT22 cells induced by OGD/R. They found that NPGE treatment could reduce neurologic impairment, reduce infarct volume, alleviate brain edema, and promote the survival of OGD/R-induced HT22 cells in ischemic stroke mice. At the molecular level, NPGE treatment relieved neuron iron death by upregulating GPX4 levels, reducing ROS and MDA, reducing excessive accumulation of Fe^2+^, improving glutathione levels, and SOD activity. In addition, NPGE treatment inhibited neuroinflammation by downregulating the levels of IL-1β, IL-6, TNF-α, NLRP3, and HMGB1. Simultaneously, NPGE treatment alleviated Erastin-induced HT22 cell iron death and inflammation. Furthermore, NPGE upregulated the expression of NRF2 and HO-1, promoting NRF2 translocation to the cell nucleus. Lastly, they also used the NRF2 inhibitor brusatol to verify that the NRF2/HO-1 signaling pathway mediated NPGE’s anti-iron cell proliferation and anti-inflammatory properties. In summary, their research results demonstrated the protective effects of NPGE and emphasized its therapeutic potential as a component of a drug for treating ischemic brain reperfusion injury.

The dried root of the medicinal plant *Astragalus membranaceus* is commonly used as a traditional Chinese medicine and has been widely used in clinical practice for the treatment of stroke, cerebral ischemia, qi deficiency, and hypertension (Fu et al., 2014; Chen et al., 2020; Zhang et al., 2021). The Bu Yang Huan Wu Tang, which has been used in traditional Chinese medicine to treat stroke for more than 200 years, has a significant effect on cerebral ischemia, with *A. membranaceus* as the main ingredient. Therefore, Chen J *et al.* (Chen et al., 2022) studied the regulation of *A. membranaceus* on transmembrane iron transport proteins and iron-related factors in rat cerebral ischemia reperfusion injury. They established a rat cerebral ischemia reperfusion injury model by using MCAO to block the blood supply of the bilateral cerebral middle cerebral artery in two male SD rats. Subsequently, they explored the regulation of *A. membranaceus* on iron transmembrane transport under conditions of cerebral ischemia reperfusion injury. Next, they used TTC staining to measure the infarct area, H&E staining to observe the histological structure of the brain tissue, and Nissl staining to assess neuronal damage. Then, they observed iron transport proteins, including ferritin (Fn), ferritin heavy chain (FHC), ferritin light chain (FLC), transferrin (Tf), transferrin receptors (TfR), DMT1, L-type calcium channels (LTCC), transient receptor potential canonical 6 (TRPC6), and FPN1, by immunohistochemistry and western blotting. Western blotting was used to detect the expression of membrane sodium-dependent cystine/glutamate reverse transport protein system Xc (System Xc) light chain subunit (XCT) and heavy chain subunit (SLC3A2), GPX4, nuclear factor erythroid 2-related factor (NFE2L2), HO-1, and iron-responsive element-binding protein 2 (IREB2) in rat brain tissue. The results showed that *A. membranaceus* reduced the infarct area and neuronal damage in the rat model after surgery. Similarly, *A. membranaceus* treatment could regulate the expression of iron transport proteins. Therefore, *A. membranaceus* was able to reduce the expression of Fn, FHC, FLC, Tf, TfR, DMT1, and TRPC6 in rats after cerebral ischemia reperfusion injury, and increase the expression of FPN1 through a Tf/TfR-independent pathway. This study suggests that *A. membranaceus* stimulation inhibits the iron death process by regulating key iron death factors, such as XCT, SLC3A2, GPX4, NFE2L2, HO-1, and IREB2. In summary, the root of *A. membranaceus* regulates transmembrane iron transport and iron death, thereby improving cerebral ischemia-reperfusion injury.

### 4.5 Metabolites alleviate cerebral stroke ischemia-reperfusion injury by regulating mitochondrial autophagy

Increasing evidence indicates that abundant metabolites in medicinal plants can mitigate cerebral stroke ischemia-reperfusion injury through regulating mitochondrial autophagy (Pineda-Ramírez et al., 2020; Ahsan et al., 2021). Ligustalactylone (LIG) is a natural compound extracted from *Chinese angelica* and *ligusticum*, which exhibits neuroprotective activity after cerebral ischemia-reperfusion injury. As reported by Mao ZG *et al.* (Mao et al., 2022), using MCAO/R as an animal model and OGD/R as an *in vitro* model, they investigated the neuroprotective effects of LIG on MCAO/R rats by performing neurobehavioral scoring, TTC staining, and HE staining. They reflected mitochondrial function by measuring the levels of ROS, MMP, and Na-K-ATPase activity and observed mitochondrial autophagy under transmission electron microscopy and fluorescence microscopy. Western blot analysis was used to examine the protein expression changes in mitochondrial autophagy mediated by PINK1/Parkin. Subsequently, cell transfection was employed to explore the specific mechanism of LIG neuroprotection and mitochondrial autophagy. The results showed that LIG enhanced mitochondrial function *in vivo* and *in vitro* by promoting mitochondrial autophagy, thereby alleviating cerebral ischemia-reperfusion injury. However, PINK1 deficiency and midivi-1 (a mitochondrial division inhibitor) further exacerbated ischemia-induced brain damage, mitochondrial dysfunction, and neuron injury, thereby eliminating the enhanced mitochondrial autophagy under cerebral ischemia-reperfusion injury. This study indicates that LIG can improve neuron damage in ischemic stroke by promoting mitochondrial autophagy through PINK1/Parkin, and targeting PINK1/Parkin-mediated mitochondrial autophagy and LIG therapy may be a promising therapeutic strategy for ischemic stroke.

Studies have shown that oxidative stress plays a crucial role in cerebral ischemia-reperfusion injury. Artemisinin (ART) is a natural metabolite extracted from medicinal plant *Artemisia annua*. Recent research indicates that ART not only has a potent antimalarial effect (Chaturvedi et al., 2010), but also exhibits antioxidant stress activity (Ahmed-Laloui et al., 2022). As reported by Jiang MH *et al.* (Jiang et al., 2022), they constructed an *in vitro* model of cerebral ischemia-reperfusion injury by using OGD/R. Subsequently, they assessed cell damage by using CCK-8 and LDH release, and evaluated the oxidative stress-induced damage and ART’s protective effects by using measurements of ROS, MDA, SOD, GSH, and MMP. The results showed that OGD/R treatment exacerbated oxidative stress damage, while ART reversed the effects of OGD/R, suggesting that autophagy might be closely related to oxidative stress. To confirm whether the antioxidant stress effects of ART were related to PHB2-mediated autophagy, they measured the protein expression of PHB2, TOMM20, p62, and the conversion of LC3I to LC3II. They found that the protein expression of PHB2, TOMM20, p62, and the LC3II/LC3I ratio was significantly correlated with OGD/R treatment. After OGD/R treatment, the colocalization of PHB2 and LC3, TOMM20 and LC3 decreased, and ART reversed these changes. Silencing PHB2 reduced the protective effects of ART on OGD/R-induced oxidative stress damage, as well as the protein expression of PHB2, TOMM20, and LC3II/LC3I and the colocalization of PHB2 and LC3B, TOMM20 and LC3B. Subsequently, they found that ART increased the conversion of LC3I to LC3II after chloroquine-induced inhibition of the lysosomal pathway, silenced PHB2, thereby inhibiting the conversion of LC3I to LC3II and impairing mitochondrial autophagy.This study demonstrates that ART alleviates OGD/R-induced oxidative stress damage through PHB2-mediated mitochondrial autophagy in human neuroblastoma SH-SY5Y cell line, providing new insights for the treatment of oxidative damage induced by cerebral ischemia-reperfusion in clinical practice.

Jionoside A1, a substance found in the traditional Chinese herb *Radix Rehmanniae Praeparata*, may possess neuroprotective properties. As reported by Yu XY *et al.* (Yu et al., 2023), they constructed *in vitro* and *in vivo* models by using OGD/R and temporary middle cerebral artery occlusion (tMCAO), respectively, and intervened with Jionoside A1 treatment and small interfering RNA (siRNA) to reduce Nix expression. The results showed that Jionoside A1 could alleviate stroke ischemia-reperfusion injury by promoting Nix-mediated mitochondrial autophagy. This study provides new experimental information for the research on ischemic stroke ischemia-reperfusion injury and lays a certain theoretical basis for its application.

Ginsenoside compound K (GCK) is a major active metabolite in *ginseng* and has shown good safety and bioavailability in clinical trials, possessing neuroprotective effects on cerebral ischemic stroke (Oh and Kim, 2016). However, its potential role in preventing cerebral stroke ischemia-reperfusion injury remains unclear. Huang QX *et al.* (Huang et al., 2023) confirmed through *in vitro* and *in vivo* models that GCK pretreatment could alleviate Drp1 mitochondrial translocation, mitochondrial autophagy, mitochondrial apoptosis, and neuronal bioenergetic imbalance, thus inhibiting stroke ischemia-reperfusion injury. Furthermore, GCK treatment could decrease the binding affinity of Mul1 and Mfn2, inhibit Mul1/Mfn2-mediated ubiquitination and degradation, and increase the protein level of Mfn2 in cerebral ischemia-reperfusion injury. In summary, these data indicate that GCK may be a promising therapeutic agent that can antagonize stroke ischemia-reperfusion injury through Mul1/Mfn2-mediated mitochondrial dynamics and bioenergetics.

### 4.6 Metabolites alleviate cerebral stroke ischemia-reperfusion injury by regulating ferroptosis

Ischemic stroke is a complex brain disease regulated by multiple cell death processes, including apoptosis, autophagy, and ferroptosis. *β*-Caryophyllene (BCP) is a natural bicyclic sesquiterpene rich in *essential oils* and has been shown to have potential pharmacological benefits in various diseases, including ischemic stroke (Fidyt et al., 2016; Yang et al., 2017; Scandiffio et al., 2020). As reported by Hu QW *et al.* (Hu et al., 2022), ferroptosis was involved in the process of ischemia-reperfusion-induced neural damage, indicating that this novel cell death might provide new therapeutic options for the clinical treatment of ischemic stroke. *In vivo* studies have demonstrated that BCP improves neurological scoring, infarct volume, and pathological characteristics after MCAO/R surgery. Furthermore, BCP significantly enhanced NRF2 nuclear translocation, activated the NRF2/HO-1 pathway, and prevented ferroptosis. Interestingly, similar results were obtained *in vitro*, where BCP reduced ROS generation and iron accumulation induced by OGD/R. Notably, the NRF2 inhibitor ML385 reversed the neuroprotective effects of BCP. These data indicate that ferroptosis plays a crucial role in cerebral ischemia-reperfusion injury, and this is the first time that a significant neuroprotective effect of BCP in reducing ischemic stroke damage has been discovered, which is closely related to the regulation of ferroptosis, possibly involving the activation of the NRF2/HO-1 axis.

Carthamin yellow (CY) is a flavonoid metabolite extracted from *safflower*, which has been shown to alleviate myocardial ischemia and reperfusion injury. However, it remains unclear whether CY can improve ischemic stroke. Guo HH *et al.* (Guo et al., 2021b) investigated the preventive effect of CY on experimental ischemic stroke using MCAO model rats. They found that after 2a0weeks of CY treatment, the neurological deficit score, brain water content, and infarct area were reduced, and MAP-2 immunoreactivity in the cerebral cortex was increased. Furthermore, CY treatment resulted in the inactivation of the cortical NF-*κ*B/NLR family pyrin domain containing three inflammatory signaling pathways, as well as decreased serum levels of TNF-*α*, IL-1*β*, and IL-6. Additional studies revealed that CY treatment inhibited the accumulation of Fe^2+^ and reactive oxygen species, as well as reversed the expression levels of acyl-CoA synthetase long chain family member 4, transferrin receptor 1, glutathione peroxidase 4, and ferritin heavy chain 1 protein in the brain. CY treatment also reversed the levels of glutathione, superoxide dismutase, and malondialdehyde in the serum. In conclusion, this study demonstrates that CY can protect rats from ischemic stroke damage, which may be achieved through alleviating inflammation and ferroptosis.

Baicalein, a major bioactive metabolite isolated from the root bark of the medicinal plants *Scutellaria baicalensis*, has been investigated for its potential role in ischemic stroke. Li M *et al.* (Li et al., 2022a) studied the potential effects of baicalein on cerebral ischemia-reperfusion injury using HT22 cells induced by OGD/R, tMCAO mice, and RSL3-simulated HT22 cells. They found that baicalein increased the viability of OGD/R-treated HT22 cells in the tMCAO model mice and significantly improved cerebral ischemia-reperfusion injury. Additionally, baicalein decreased the levels of iron, lipid peroxidation, and morphological features of ferroptosis in the brain tissue of tMCAO model mice, indicating that baicalein can alleviate cerebral ischemia-reperfusion injury *in vitro* and *in vivo* by inhibiting ferroptosis. Further studies confirmed the inhibitory activity of baicalein on ferroptosis in RSL3-stimulated HT22 cells. The results of western blotting showed that baicalein inhibited ferroptosis by regulating the expression levels of GPX4, ACSL4, and ACSL3 in OGD/R-treated cells, tMCAO mice, and RSL3-stimulated HT22 cells. These data suggest that baicalein can reverse cerebral ischemia-reperfusion injury by resisting ferroptosis, possibly through the regulation of the GPX4/ACSL4/ACSL3 axis, which reflects the potential of baicalein as a therapeutic agent for cerebral ischemia-reperfusion injury.

Reypolysaccharide A is derived from medicinal plant *Radix Rehmanniae Praeparata*, which is widely used as an important component of a variety of medicinal plants in China, and is mainly used for the treatment of cerebral arteriosclerosis, stroke and senile dementia (Zhang et al., 2008). Recent studies have shown that Reypolysaccharide A can improve memory and neuronal damage. As Fu C *et al.* (Fu et al., 2022) first revealed the DEP in patients with ischemic stroke by using the RayBio protein array. They then established a cognitive impairment model in rats using a 14-day MCAO treatment and intraperitoneal injection of 80 mg/kg Reypolysaccharide A, as well as exposing SH-SY5Y cells to H_2_O_2_ for 24 h and treating them with 80 μM Reypolysaccharide A for 24 h. Subsequently, they assessed the neuroprotective effects of Reypolysaccharide A by detecting the infarct volume *in vivo* (TTC staining), the neurological deficit (Garcia score) and learning memory (morris water maze test), and cell viability *in vitro* (CCK-8 and LDH). Meanwhile, the biochemical methods were used to detect the activities of SOD, MDA and myeloperoxidase (MPO) in rats, as well as the levels of GSH, oxidized glutathione (GSSG) and nicotinamide adenine dinucleotide phosphate (NADPH) in cell. And the DCFH-DA was used to measure the level of ROS. Finally, the expression levels of MPO, PI3K, p-PI3K, Akt, p-Akt, HO-1, Nrf 2, SLC7A11, and GPX 4 proteins in the rat cerebral cortex were detected by the western blotting technology. The results of the *in vivo* study showed that compared with the model group, the cognitive impairment and neurological deficits in the Reypolysaccharide A treatment group were significantly improved, and the cerebral infarction of MCAO rats was reduced. In addition, the results of the *in vivo* study showed a significant increase in cell viability and a decrease in toxicity induced by the H_2_O_2_ + Reypolysaccharide A group. Further studies showed that the expressions of p-PI3K, p-Akt, Nrf2, HO-1 and SLC7A11 proteins in the Reypolysaccharide A-treated group were significantly higher than those in the model group. These data suggest that Reypolysaccharide A has neuroprotective effects and improves cognitive impairment after cerebral ischemia by inhibiting ferroptosis and activating the PI3K/AKT/Nrf2 and SLC7A11/GPX4 signaling pathways, and these findings also provide valuable insights into the pathogenesis and therapeutic targets of ischemic stroke.

## 5 Conclusion and future directions

Mitochondrial autophagy and ferroptosis are newly discovered types of programmed cell death associated with the development of cerebral ischemia-reperfusion injury. Pharmacological activators of mitochondrial autophagy and pharmacological inhibitors of ferroptosis can alleviate early brain damage caused by ischemia-reperfusion. However, when the disease progresses to the middle and late stages, it inevitably increases the load on the organs and triggers excessive and persistent mitochondrial autophagy and ferroptosis in the body, resulting in the production of ROS and inflammation, further causing cortical cell death, exacerbating the destruction of the blood-brain barrier, and promoting the entry of more bacteria or viruses into the brain, ultimately exacerbating the malignant cycle of the disease. Therefore, specifically inhibiting the processes of mitochondrial autophagy and ferroptosis may improve middle and later stage cerebral ischemia-reperfusion injury. It is thus particularly important to regulate the processes of mitochondrial autophagy and ferroptosis reasonably based on the disease development stage. Although many advances have been made in understanding the mechanisms and roles of mitochondrial autophagy and ferroptosis in brain diseases, further clinical application of targeted therapy for these processes is still in the early stages. Their specific roles remain to be investigated in the context of brain diseases, including many that were not covered in this review (such as cerebral arteriosclerosis, epilepsy, Alzheimer’s disease and so on). Therefore, we believe that more research, including cell experiments, animal experiments, and clinical studies, is needed to understand the roles of different cell death processes in brain diseases more thoroughly, thus providing new clinical treatment methods for brain diseases. Interestingly, many brain diseases are associated with one or more cell death processes, including other patterns not mentioned in this review, such as necroptosis, endoplasmic reticulum stress, oxidative stress, pyroptosis, cupric death, and disulfide death. Therefore, further research is needed to determine the specific cell death mechanisms involved in brain diseases, thus developing treatment approaches relevant to the disease context. Future studies should focus on the interplay between cell death processes in brain diseases.

Given the complex etiology of cerebral ischemia-reperfusion injury, single-targeted therapy based on mitochondrial autophagy or ferroptosis is often unsatisfactory. However, medicinal plants have unique advantages in treating cerebral ischemia-reperfusion injury. This can be attributed to two main aspects: Firstly, the mechanisms of mitochondrial autophagy and ferroptosis involve multiple signaling pathways (including PINK1/Parkin, AMPK, E-cadherin/NF2/Hippo/YAP, HIF-2α/HILPDA, and so on), and it is sometimes difficult for a single-targeted drug to address all pathways. Fortunately, increasing evidence shows that many medicinal plants and their active metabolites can not only act through multiple targets but also consider multiple targets (such as NIX, BNIP3, FUNDC1, GPX4, FSP1, and so on) in cerebral ischemia-reperfusion injury, such as mitochondrial autophagy and ferroptosis. Secondly, medicinal plants and their active metabolites have been proven to be safe, especially with fewer toxic effects on liver and kidney damage compared to western medications. For instance, traditional Chinese herbal compound preparations such as Taohong Siwu decoction, Xiao-Xu-Ming decoction, and compound tongluo decoction have been used for thousands of years in China, and their clinical efficacy is relatively optimistic. It is worth noting that in recent years, many researchers have attempted to isolate active ingredients from clinically effective herbal formulas by chemical methods and apply them to cell or animal experiments to elucidate their molecular mechanisms. To date, research on the regulation of mitochondrial autophagy and ferroptosis in herbal plants related to cerebral ischemia-reperfusion injury still has many limitations, and the study of mitochondrial autophagy and ferroptosis regulation in cerebral ischemia-reperfusion injury is still in the observational research stage, lacking deeper exploration of mechanisms. Therefore, more standardized and larger-scale clinical studies are needed to promote the wider acceptance of medicinal plant therapy in patients with cerebral ischemia-reperfusion injury and hope that these medicinal plants and their active metabolites can withstand experimental scrutiny and bring benefits to patients with cerebral ischemia-reperfusion injury as soon as possible.
